# Effects of glucose and lactate on *Streptococcus mutans* abundance in a novel multispecies oral biofilm model

**DOI:** 10.1128/spectrum.03713-23

**Published:** 2024-02-20

**Authors:** Jay S. Sangha, Paul Barrett, Thomas P. Curtis, Aline Métris, Nicholas S. Jakubovics, Irina D. Ofiteru

**Affiliations:** 1 School of Engineering, Newcastle University, Newcastle upon Tyne, United Kingdom; 2 Safety and Environmental Assurance Centre, Unilever, Colworth Science Park, Sharnbrook, United Kingdom; 3 School of Dental Sciences, Faculty of Medical Sciences, Newcastle University, Newcastle upon Tyne, United Kingdom; The Ohio State University College of Dentistry, Columbus, Ohio, USA

**Keywords:** *Streptococcus mutans*, chemically defined medium, *in vitro* biofilm model, CDC biofilm reactor, pH, fluorescence *in situ* hybridization (FISH), qPCR

## Abstract

The oral microbiome plays an important role in protecting oral health. Here, we established a controlled mixed-species *in vitro* biofilm model and used it to assess the impact of glucose and lactate on the ability of *Streptococcus mutans*, an acidogenic and aciduric species, to compete with commensal oral bacteria. A chemically defined medium was developed that supported the growth of *S. mutans* and four common early colonizers of dental plaque: *Streptococcus gordonii*, *Actinomyces oris*, *Neisseria subflava*, and *Veillonella parvula*. Biofilms containing the early colonizers were developed in a continuous flow bioreactor, exposed to *S. mutans*, and incubated for up to 7 days. The abundance of bacteria was estimated by quantitative polymerase chain reaction (qPCR). At high glucose and high lactate, the pH in bulk fluid rapidly decreased to approximately 5.2, and *S. mutans* outgrew other species in biofilms. In low glucose and high lactate, the pH remained above 5.5, and *V. parvula* was the most abundant species in biofilms. By contrast, in low glucose and low lactate, the pH remained above 6.0 throughout the experiment, and the microbial community in biofilms was relatively balanced. Fluorescence *in situ* hybridization confirmed that all species were present in the biofilm and the majority of cells were viable using live/dead staining. These data demonstrate that carbon source concentration is critical for microbial homeostasis in model oral biofilms. Furthermore, we established an experimental system that can support the development of computational models to predict transitions to microbial dysbiosis based on metabolic interactions.

**IMPORTANCE:**

We developed a controlled (by removing host factor) dynamic system metabolically representative of early colonization of *Streptococcus mutans* not measurable *in vivo*. Hypotheses on factors influencing *S. mutans* colonization, such as community composition and inoculation sequence and the effect of metabolite concentrations, can be tested and used to predict the effect of interventions such as dietary modifications or the use of toothpaste or mouthwash on *S. mutans* colonization. The defined *in vitro* model (species and medium) can be simulated in an *in silico* model to explore more of the parameter space.

## INTRODUCTION

The human microbiome plays an important role in health and disease (1, 2) and is subjected to perturbations, for example, via the application of beauty and personal care products (3). After the colonic microbiome, the oral microbiome is the second most diverse microbial community colonizing the human body and consists of microbes in saliva and on surfaces of hard and soft tissues in the mouth. Microbial dysbiosis in the biofilms growing on tooth surfaces is associated with the most common bacterial diseases in humans: dental caries and periodontitis (4). Caries development is favored by dietary factors such as high-frequency consumption of sugars (5). Acidogenic oral bacteria ferment sugars to produce organic acids such as lactic, acetic, and propionic acids that alter the local environment by decreasing the local pH. A lower pH gives a competitive advantage to aciduric and acidogenic species such as *Streptococcus mutans*, *Scardovia wiggsiae*, or members of the *Lactobacillaceae* family (6). These species, in turn, produce more acids that demineralize the enamel and dentine of the teeth (7). This positive feedback loop stimulates caries appearance unless it is balanced by health-maintaining mechanisms, such as buffering by saliva (8), low-sugar diet, fluoride intake, and mechanical removal of dental biofilms by regular tooth brushing or flossing (9).

Although the salivary microbiome varies across individuals, an analysis of 47 studies using 16S rRNA gene sequencing demonstrated that there is a core microbiome with 68 operational taxonomic units mapped to species or species-level equivalents that are detectable in almost everyone (10). Of these, members of the genera *Streptococcus*, *Haemophilus*, *Prevotella*, *Neisseria*, *Rothia*, *Veillonella*, and *Fusobacterium* tend to be present consistently in high relative abundance and can be observed within multispecies aggregates in saliva (10, 11). When these aggregates attach to a tooth surface, certain bacteria within them start to grow rapidly and provide the foundations of dental biofilms. These are known as pioneer colonizers and include members of the genera *Streptococcus*, *Actinomyces*, *Neisseria*, and *Veillonella* (11, 12). The pioneer colonizers do not cause damage directly and are thought to contribute to the resilience against oral disease (6, 13). For example, the presence of alkali-generating microbes in dental biofilms may be as important for caries prevention as the absence of high levels of acidogenic species (14). It is, therefore, important to study the factors with potential to modify the composition and resilience of the oral microbiota and to understand the relationship between the members of the community and the conditions in which a pathobiont becomes dominant (dysbiosis). This understanding is critical for the development of new approaches to maintain oral health and for providing a timely and sensitive assessment of the safety of novel interventions (2).

Microbiome composition is most often measured in clinical experiments, which are the gold standard for evaluating methods to control oral biofilms (15). Nevertheless, clinical studies are very costly and hampered by the significant effects of host factors, including age, sex, diet, and saliva, which can confound the results (16, 17). In addition, since the mouth is a semi-open system, it is challenging to effectively control the influencing factors and to quantify their impact. Therefore, when assessing the effect of intervention, *in vitro* systems can help to evaluate safety and eliminate the host variability factor (18). Despite their inevitably simplified nature, these systems provide a useful model for hypothesis-driven research (19). A number of mixed-species biofilm models have been established to investigate the development of oral biofilms, including those associated with dental caries (15). For example, biofilms containing *Actinomyces naeslundii*, *Streptococcus oralis*, and *S. mutans* have been used to investigate *S. mutans* adhesion and gene and protein expression during biofilm development (20, 21). To study metabolic interactions and carbohydrate utilization, biofilms containing up to 19 different species were developed in a chemically defined medium under static conditions (22). However, static biofilms do not mimic the continual replenishment of nutrients that occurs in the oral cavity.

The aim of this study was to develop an *in vitro* system where bacterial metabolism, such as sugar consumption and acid production, can be quantified and where shifts to dysbiosis can be observed by monitoring the relative abundance of *S. mutans* to pioneer colonizers. This required the use of a chemically defined medium and a representative community of co-existing bacteria that remain stable under various relevant conditions, thus striking a balance between complexity and representativity.

We selected a cohort of four pioneer colonizing oral bacteria (*Streptococcus gordonii*, *Actinomyces oris*, *Neisseria subflava*, and *Veillonella parvula*) that are generally associated with health (12). In addition, *S. mutans* was included in biofilms as an aciduric and acidogenic species that has been associated with the development of dental caries. Bacteria were grown in a continuous flow system using the Centre for Disease Control (CDC) coupon biofilm bioreactor and in a novel chemically defined medium developed to support the growth of all five species, with different concentrations of glucose and lactic acid. We hypothesized that modulating the concentrations of glucose and lactic acid would affect the biofilm development and the ability of *S. mutans* to dominate biofilms. This model can be exploited further in conjunction with mathematical modelling to characterize factors leading to a cariogenic shift of the microbiome.

## RESULTS

### Development of a novel chemically defined medium

**Fig 1:**
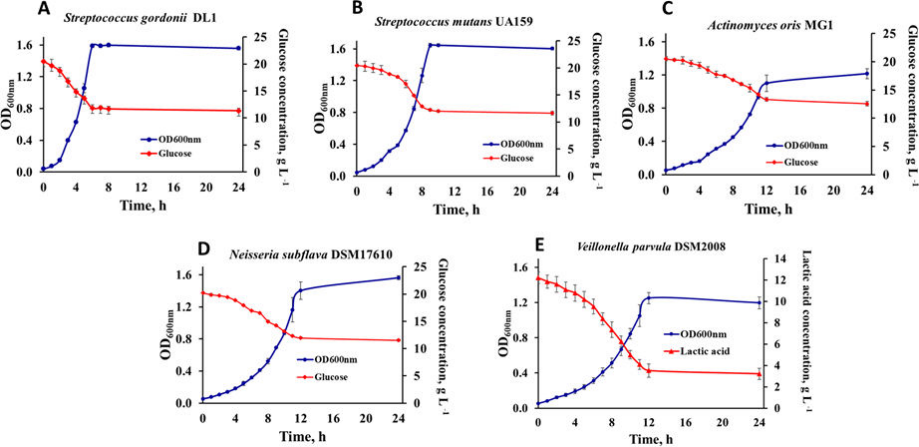
Growth curves and substrate consumption for the individual species on AFMC. (**A**) *Streptococcus gordonii* DL1, (**B**) *Streptococcus mutans* UA159, (**C**) *Actinomyces oris* MG1, (**D**) *Neisseria subflava* DSM17610, and (**E**) *Veillonella parvula* DSM2008. All species were grown on AFMC with 20 g L^−1^ glucose and 12.1 g L^−1^ lactic acid. Data points show the average OD_600_ and substrate concentration across biological triplicates all with three technical triplicates. Error bars represent the standard deviation from the mean (*n* = 3).

Initially, a novel chemically defined medium was developed based on the Fortified M1 medium with Citrate (FMC) medium described by Terleckyj et al. (23) that sustained the growth of the five chosen bacterial species. Unamended FMC containing 20 g L^−1^ D-glucose supported the growth of *S. gordonii*, *S. mutans*, and *N. subflava* but not *A. oris* or *V. parvula*. Essential nutrients for *A. oris* and *V. parvula* were identified from literature searching. The addition of L-cysteine (1 g L^−1^), inositol (2 mg L^−1^), thioctic acid (0.1 mg L^−1^), oleic acid (2 mg L^−1^), and pimelic acid (0.1 mg L^−1^) enabled the growth of *A. oris*. Further supplementation with putrescine (1 mg L^−1^) additionally supported *V. parvula* growth. The base medium without the major carbon sources glucose and lactic acid was termed “amended FMC” (AFMC) medium, and the composition is given in Table A1. For monospecies growth experiments, the AFMC medium was supplemented with 20 g L^−1^ glucose as the preferred primary carbon source for *S. gordonii*, *S. mutans*, *A. oris*, and *N. subflava* (24, 25) and 12.1 g L^−1^ lactic acid, as the primary carbon source for *V. parvula*, which cannot metabolize glucose (26). With these carbon sources, AFMC supported the growth of each of the five species (see Fig. 1). Each species was able to grow for at least 40 generations in AFMC (data not shown), and the pH after 48 h for each species ranged from 4.94 (*S. mutans*) up to 5.98 (*V. parvula*).

To inform the sequence of inoculation in the CDC bioreactor model and to verify that the selected flow rate would not lead to washout of cells, we estimated the Monod kinetic parameters (maximum specific growth rate and substrate affinity constant) of the five species in AFMC (Table 1). The initial pH of all the bacterial cultures was adjusted to neutral. The estimated values of the kinetic parameters confirmed previous literature reports that *A. oris* is a slower grower (27) and therefore, has to be inoculated first in order to get established in the bioreactor. The maximum specific growth rates for *S. mutans* and *S. gordonii* were similar and show that they can compete for growth (27). However, *S. mutans* had a smaller estimated affinity constant, indicating that it can thrive at lower glucose concentrations. It is important to note that the kinetic parameter estimations are valid only for neutral pH and do not consider the changes induced by the acid tolerance response when *S. mutans* is known to have a competitive advantage (28).

**TABLE 1:** Kinetic parameters measured on AFMC for the bacterial species included in the study

Bacterial species	Primary carbon source	*µ*_max_ (h^−1^)	*K_s_* (g·L^−1^)
*S. gordonii* DL1	Glucose	0.492	1.88
*S. mutans* UA159	Glucose	0.406	1.00
*A. oris* MG1	Glucose	0.227	1.40
*N. subflava* DSM17610	Glucose	0.261	1.00
*V. parvula* DSM2008	Lactic acid	0.246	2.42

### Development of the five-species model community in the CDC coupon bioreactor (bulk and biofilm)

We investigated the potential of the selected species to develop a stable microbial community when cultivated in a continuous flow CDC coupon bioreactor system (working volume 350 mL). All species were cultivated in the AFMC medium, supplemented with glucose and lactic acid. Based on the kinetic parameter estimation, the inoculation sequence started with *A. oris* on Day 0 and *S. gordonii*, *N. subflava*, and *V. parvula* on Day 1, followed by *S. mutans* on Day 2. We selected a flow rate of 0.4 mL min^−1^, a value that falls within the range of normal unstimulated salivary flow (0.3–0.5 mL min^−1^) (29). This ensured that the dilution rate in the system (0.07 h^−1^) is significantly lower than the washout dilution rate calculated for the five species (which ranged from 0.2 h^−1^ for *V. parvula* to 0.45 h^−1^ for *S. gordonii*), allowing all of them to get established in the system.

We evaluated the synthetic community development for three distinct combinations of initial primary carbon source concentrations (see Table 2), namely, Reactor Experiment 1 (RE 1) with high glucose and high lactic acid, RE 2 with low glucose and high lactic acid, and RE 3 with low glucose and low lactic acid.

**TABLE 2:** Substrate concentrations and pH for the three REs (average ±SD over three replicates)

Experiment	Inlet and initial glucose conc. (g·L^−1^)	Final glucose conc. (g·L^−1^)	Inlet and initial lactic acid conc. (g·L^−1^)	Final lactic acid conc. (g·L^−1^)	Bulk initial pH (Day 0)	Bulk final pH (Day 9)
RE 1	21.85 ± 0.94	9.02 ± 0.76	11.61 ± 1.22	3.97 ± 1.23	6.76	5.21
RE 2	2.09 ± 0.12	0.04 ± 0.01	12.46 ± 0.32	5.53 ± 0.39	6.81	6.01
RE 3	1.98 ± 0.76	0.04 ± 0.01	2.61 ± 0.10	0.40 ± 0.02	6.95	6.24

For the experiments with high glucose concentration (RE 1), the pH stabilized below 5.5 and remained there until the experiment was terminated at Day 9 (Table 2). In the experiments with low glucose concentration (RE 2 and RE 3) the bulk pH remained above 5.5.

**Fig 2:**
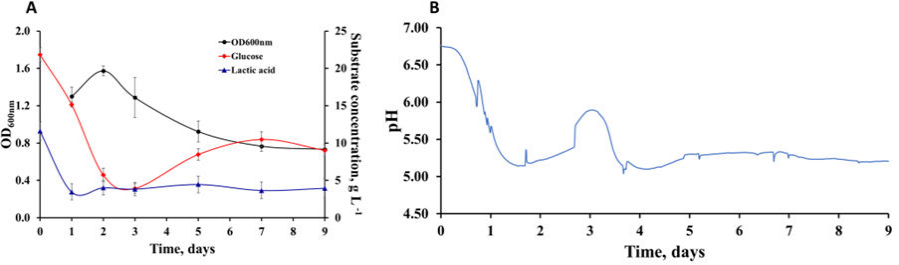
Bulk measurements in the CDC coupon bioreactor during the RE 1 (average over three replicates). (**A**) Cell density (OD_600nm_), concentration of glucose and lactic acid; (**B**) pH.

**Fig 3:**
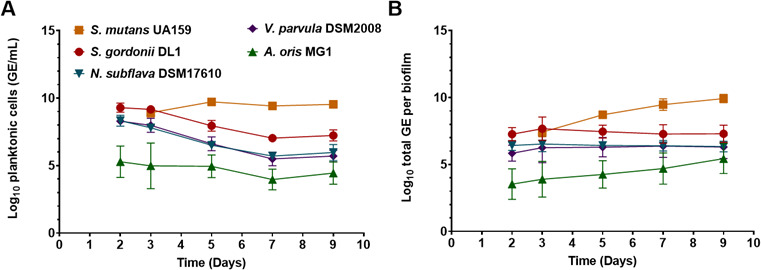
Species absolute numbers in the CDC reactor during the RE 1, measured by TaqMan qPCR analysis with species-specific primers and probes. (**A**) Bulk samples; (**B**) hydroxyapatite coupons samples. Mean and SD from three replicates are shown. GE, genome equivalents.

In each of the three REs, a stable microbial community was achieved after a period of 9 days. Here, we showcase the results of RE 1, with bulk measurement (OD_600nm_, substrate concentration and pH) included in Fig. 2, and cell absolute counts in the bulk and in the biofilm formed on the hydroxyapatite coupons shown in Fig. 3 (A and B, respectively). Equivalent data from RE 2 to RE 3 are included in Figures A1–A4. In all the experiments, all species successfully established in the bioreactor by Day 2 and were identified in the synthetic community formed. However, at high glucose concentration, the introduction of *S. mutans* on Day 2 led to a subsequent decrease in population sizes for all the other four species, in both the bulk and the coupon biofilm (Fig. 3). On Day 9, at the end of the experiment, *S. mutans* was the dominant species in the system.

**Fig 4:**
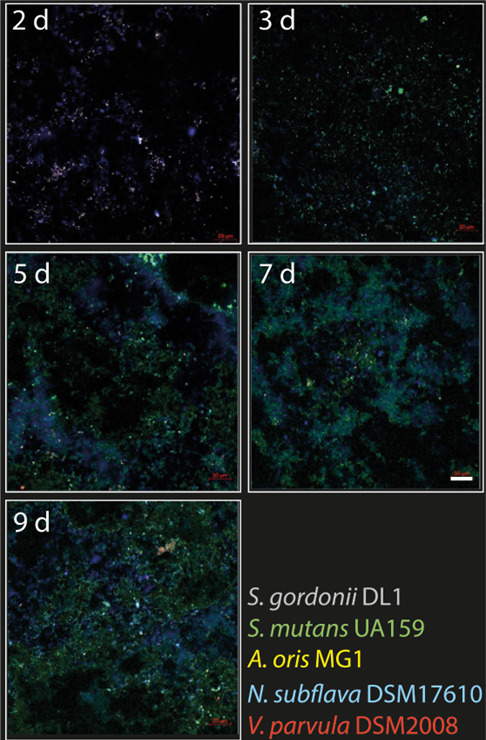
Selected FISH-CLSM images showing the accumulation of multispecies biofilms over time in RE1. Species were labelled with unique FISH probes, and signals were separated by spectral imaging. Images are labelled in days (d) after inoculation of the first species (*A. oris* MG1) at Day 0. *S. mutans* UA159 was not inoculated until Day 2 and is not visible in the first panel (2 d). After this, *S. mutans* UA159 levels gradually increased in biofilms, and they were the dominant species from Day 5 onward. Bar = 20 µm.

The co-existence of the bacterial species in the biofilm and the dominance of *S. mutans* at high glucose concentration (RE 1) were confirmed also through fluorescence *in situ* hybridization (FISH) and visualized through confocal laser scanning microscopy (CLSM); see Fig. 4. A more balanced composition of the biofilm was visible at low glucose and low lactic acid concentrations (RE 3; Figure A5), confirming the quantitative PCR (qPCR) results (Figure A4).

### Glucose concentration dictates *S. mutans* levels in the biofilm

**Fig 5:**
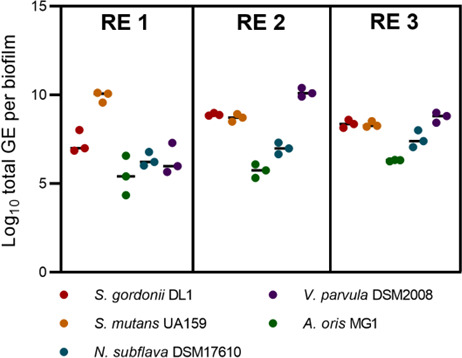
Total cells in the biofilm (qPCR) at Day 9, for all three experimental conditions. *S. mutans* UA159 dominated at high glucose concentration (RE 1), whereas *V. parvula* DSM2008 dominated in high lactate (RE 2). A more balanced community was observed at low concentrations of glucose and lactate (RE 3). GE, genome equivalents. Bars show mean values (*n* = 3).

The concentration of each species in biofilms under the three reactor conditions was assessed at Day 9 by qPCR (Fig. 5). At high glucose concentration (RE 1), biofilms were dominated by *S. mutans*. There was a marked difference in the composition of biofilms in low-glucose conditions (RE 2 and RE 3). Although *S. mutans* successfully established in these biofilms, it was not the most abundant species. In RE 2 (low glucose and high lactate), there was a clear dominance of *V. parvula*, consistent with its preference for lactate as a carbon source. The conditions in RE 3 (low glucose and low lactate) resulted in a more balanced microbial community in which there was only a 315-fold difference between the least abundant (*A. oris*) and most abundant (*V. parvula*) species. In the bulk fluid of the reactor, *S. mutans* dominated in all the conditions tested (Figure A6) but at low glucose concentration (RE 2 and RE 3), *V. parvula* established as the second most abundant species and similar in concentration to *S. mutans*. The viability of cells in 9-day planktonic cultures and biofilms was assessed by live/dead staining with flow cytometry (Figure A7). In all samples, at least 75% of cells were viable, except the bulk fluid of RE 1 (42.6% viable cells).

## DISCUSSION

Here, we developed a novel oral biofilm model using a chemically defined medium to assess the impact of specific nutrients on the composition and cariogenic potential of five-species biofilms. The use of a fully defined synthetic medium is essential for constructing realistic computational models to predict potential cariogenicity during the design of novel oral care products. We have developed a synthetic medium (AFMC) that supported the growth of five different oral bacteria. Using AFMC, all five species were retained within the bulk fluid and on biofilms in a continuous flow CDC bioreactor model for up to 9 days. Varying glucose and lactate concentrations impacted the microbial composition within biofilms and affected the pH within the system. In particular, high glucose concentration resulted in a predominance of *S. mutans* within biofilms, whereas high lactate was selected for *V. parvula* biofilm growth.

Several model systems are available to study *in vitro* oral biofilms, as recently reviewed in Luo et al. (15). We have chosen the continuous flow reactor as the flow-through medium can mimic the dental biofilm exposed to saliva. By including an aerobic bacterium (*N. subflava*) to act as an oxygen scavenger, we ensured that the strict anaerobes are protected and can establish in the system, as previously reported for chemostat and biofilm experiments with dental biofilm species (30). Moreover, a recent study indicates that *N. subflava* may be a keystone member of oral microbial communities, having an impact that is disproportional to its abundance in the system (31). Combined with the use of a chemically defined medium, such a system allows control of the environmental factors and enables single parameters to be varied independently. The CDC coupon bioreactor, which has multiple biofilm sampling points, was previously used as a reproducible microcosm biofilm representative of the oral microbiome (32), but former studies used saliva and dental plaque inoculation, and the feed was with a complex medium (33). We have used a chemically defined medium that allows the modification of one parameter at the time, thus providing a powerful strategy for studying how a biofilm develops and how its structure can be disrupted. The FMC was previously used for growing *S. mutans* in monoculture (34–36), but our amended formulation supports a more complex community of early colonizers. The use of chemically defined medium for a multispecies oral biofilm model differentiates our work from previous reports (30, 37) which have used complex medium supplemented with hog gastric mucin. Our results are in agreement with this previous work, showing, in addition, that the level of glucose itself can drive the pH and *S. mutans* dominance in the biofilm and give insights into metabolic interactions. We deconvoluted the effect of metabolism of early colonizers on *S. mutans* dominance from other effects to be able to quantify the factors affecting colonization. In further experiments, to study the additional effect of metabolites on the biofilm structure, the effect of alternative energy sources such as sucrose and mucin could be assessed.

Although hog gastric mucin has been used in various formulations of “artificial saliva,” it is chemically and structurally distinct from the major human salivary mucins MG1 and MG2. Recent work has shown that different mucin glycans shape microbial communities through impacts on nutrition, aggregation, and potentially also interspecies competition (38). Modelling the contribution of mucins or other complex salivary glycoproteins to microbial biofilm formation will require consideration of these multifaceted effects on biofilms. Similarly, sucrose has an impact on oral biofilm formation beyond simple uptake and metabolism. This is due to the capacity of *S. mutans* and certain other oral bacteria to secrete glucosyltransferase and fructosyltransferase enzymes that synthesize extracellular glucans and fructans. Insoluble glucans in particular form a bulky matrix that promotes adhesion and colonization (39). In fact, the ability of *S. mutans* to produce robust biofilms from sucrose *in vitro* has led to the common use of sucrose in caries biofilm models (40). However, *in vivo* studies have shown that sucrose is not the only sugar that can promote dental caries; other sugars including glucose and maltose are also cariogenic (41).

Here, we showed that high concentrations of glucose select for the growth of *S. mutans* in biofilms in preference to early colonizers of dental plaque, including *S. gordonii*. Interestingly, the maximum specific growth rate (*µ*_max_) of *S. gordonii* in monocultures was slightly higher than *S. mutans* with glucose as the primary carbon source (0.492 h^−1^ compared with 0.406 h^−1^). However, *S. mutans* has a powerful acid adaptation response and thrives in conditions of low pH (42). It is likely that the sustained low pH in the bulk of the CDC bioreactors containing high glucose (RE 1) enabled *S. mutans* to outcompete other species. Note that the pH in the depth of the biofilm is likely to be different from the bulk due to the diffusion limitation through the biofilm matrix (43). Nevertheless, in the CDC coupon bioreactor, the bulk community is a continuous source of bacteria for the biofilm formed on the coupons.

Although *V. parvula* was included in our model, its relationship with dental caries is not entirely clear (44). *Veillonella* spp. are often elevated in the salivary microbiome of individuals with early childhood caries, and they have been shown to co-occur with certain salivary immunological markers in children with dental caries (45). *V. parvula*/*dispar* co-occurs with *S. mutans* in root caries in older patients and promotes *S. mutans* biofilm growth when co-cultured *in vitro* (46). Since *Veillonella* spp. do not produce acid, it is likely that they do not contribute directly to the acidification of caries-associated biofilms and dissolution of enamel. Instead, the utilization of lactic acid by *Veillonella* spp. likely enables continued growth of *S. mutans* in conditions where acid production is high. Here, we chose to supply lactic acid also through the reactor feed, as preliminary experiments indicated that *V. parvula* was not establishing in the community. Low glucose/high lactate conditions (RE 2) led to overgrowth of *V. parvula* in biofilms. *S. mutans* levels were not higher than *S. gordonii* in these biofilms even though *S. mutans* was more abundant in the bulk fluid (Figure A6). As *S. mutans* relies more heavily on sucrose-dependent glucans for adhesion (47) and in our experiments we have only provided glucose, integration into the biofilm may have been a rate-limiting step for *S. mutans*. In addition, *S. mutans* was deliberately inoculated into the model 2 days after establishment of the early colonizing species, which may have limited its ability to compete in biofilms in the absence of strong selection through high sugar/low pH.

The reproducibility of our *in vitro* model shows it can be used to study the conditions in which the ability of *S. mutans* to dominate within the oral biofilm is disrupted [“control without killing” (48)], thereby preventing the onset of dental caries by inducing modulations of the microbiota (49). Moreover, by using a chemically defined medium, we can complement the experiments reported here with an equivalent *in silico* model to include defined stoichiometry and the Monod kinetic parameters measured *in vitro*, as well as the pH calculation. To improve our understanding of the functions of the oral microbiome (50), we can simulate the effect of each parameter and the effect of interactions between the different species. To date, there are very few studies which connected and validated the same dental biofilm model both *in vitro* and *in silico*. The notable exceptions [e.g., Rath et al. (51)] used monospecies biofilm and validated the experiments only based on one characteristic of the overall biofilm (e.g., height), rather than biofilm composition. Our five-species system is therefore an important advancement in the field and can be used for directing safe oral hygiene product development.

## MATERIALS AND METHODS

### Bacterial strains growth and media

Five reference bacterial strains were used in this study: *S. gordonii* DL1 (Challis) (52), *S. mutans* UA159 (53), and *A. oris* MG1 (54), which were obtained from our local culture collection, and *N. subflava* DSM17610 and *V. parvula* DSM2008, which were purchased from the German Collection of Microorganisms and Cell Cultures GmbH (DSMZ). All strains were routinely cultured in THYE containing (per L) 30 g Todd Hewitt broth and 5 g Yeast Extract (Melford, Ipswich, UK). For *V. parvula*, THYE was supplemented with 1% (vol/vol) lactic acid syrup (Sigma Aldrich, Missouri, USA) to produce THYEL medium. *S. gordonii*, *S. mutans*, *A. oris*, and *V. parvula* were cultured anaerobically (80% N_2_, 10% H_2_, and 10% CO_2_) at 37°C in a Whitley DG250 cabinet (Don Whitley Scientific, Bingley, UK), while *N. subflava* was cultured aerobically in an IKA KS 400 incubator (Staufen, Germany) at the same temperature. The cells in exponential growth phase were harvested by centrifugation (3,800 × *g* at 4°C), washed, and resuspended in 20 mL chemically defined medium (AFMC) and used as inoculum for the growth experiments. The complete composition of AFMC is included in Table A1.

The growth experiment of each species in AFMC was performed in test tubes at 37°C with initial dilution 1:25 (0.2 mL inoculum to 4.8 mL medium). The AFMC was supplemented with 20 g L^−1^ glucose and 12.1 g L^−1^ lactic acid. The test tube cultures were monitored for 24 h, with hourly readings of the optical density at 600 nm (Biochrom Libra S11, Biochrom, Cambridge, UK). The Monod kinetic parameters for each species (the maximum specific growth rate and the substrate affinity constant) were estimated by linearization (55) based on the growth curves at different concentrations of glucose and lactic acid (one limiting substrate for each species).

### Substrates measurement

Glucose and lactic acid concentrations were measured using D-Glucose and D-L Lactic Acid Assay Kit (Megazyme, Ireland), respectively, according to the manufacturer’s instructions.

### Culture in CDC biofilm bioreactors

**Fig 6:**
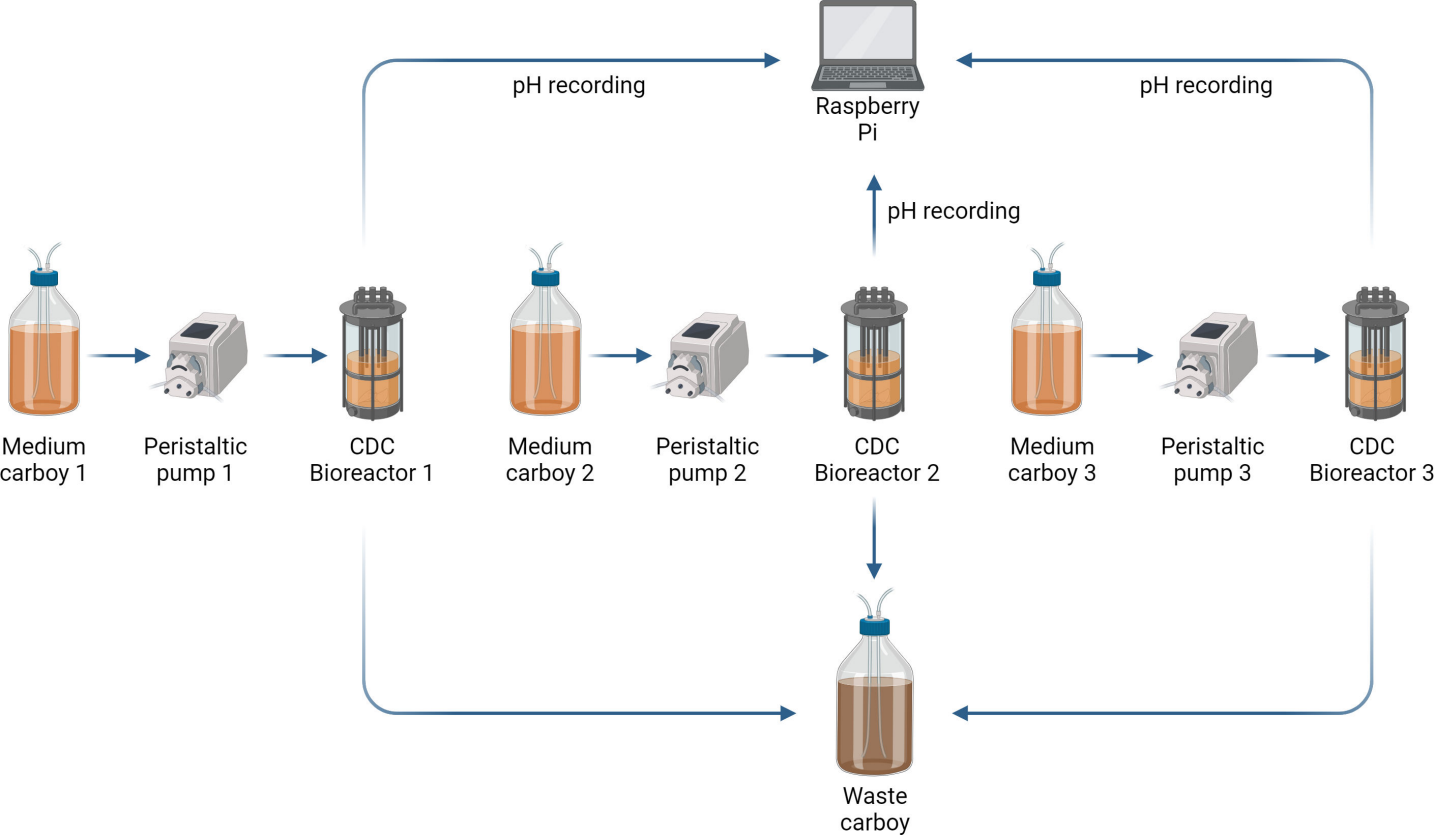
CDC reactors setup. Each reactor, kept on a hot plate to maintain temperature, had its own medium carboy, containing 6 L of AFMC, fed at a flow rate of 0.4 mL min^−1^. One port per vessel contained the pH probe, which continuously recorded the pH. The reactors were connected to a 20 L waste carboy. Figure created with BioRender.com.

The REs were run three times for each set of conditions using a CDC Biofilm Reactor (Biosurface Technologies, Montana, USA) with hydroxyapatite coupons, as per the setup schematic presented in Fig. 6. All reactor components were autoclaved at 121°C for 15 min prior to use. The reactors were maintained at 37°C using hot plates and triple-wrapped insulation and were continuously fed with 0.4 mL min^−1^ AFMC medium. The pH of the bulk was continuously measured with a F-695 autoclavable pH probe (Broadley James, California, USA) connected to a Raspberry Pi 3 (Raspberry Pi Foundation, Cambridge, UK) using Atlas software (Atlas Software Technologies, Illinois, USA). The reactors were inoculated with approximately 3.85 × 10^9^ cells from each bacterial species. Samples of the bulk culture were taken through the sampling port on the top of the reactor. The biofilm samples were collected from the hydroxyapatite coupons, which were removed from the reactors on days 2, 3, 5, 7, and 9.

### Measurement of cell concentration in bulk fluid and biofilms by qPCR

The bulk samples (1 mL, in triplicate) were centrifuged at 12,000 × *g* at 4°C. The resulting pellet was suspended in 1 mL phosphate-buffered saline (PBS), vortexed and centrifuged, and then resuspended in 1 mL PBS for further analysis. The biofilm samples were scraped off the hydroxyapatite coupons and suspended in 1 mL PBS, vortexed and centrifuged at 12,000 × *g* at 4°C, and then resuspended in 1 mL PBS. DNA extraction was performed using a DNeasy Powersoil Pro Kit (Qiagen, Hilden, Germany) according to the manufacturer’s instructions, for both bulk and biofilm samples. DNA concentration and purity were checked with NanoDrop (Thermo Fisher Scientific, MA, USA). After extraction, DNA was stored at −20°C until further analysis.

The qPCR primers and probes used, specific to each bacterial strain, are listed in Table 3 and were purchased from DNA Oligo Synthesis Services (Thermo Fisher Scientific, MA, USA). The qPCR reaction mixture for each species contained 300 nM of forward and reverse primer, 150 nM of probe, 12.5 µL of 2× Premix ExTaq Mix (Takara, Shiga, Japan), 2 mM Rox (Takara), nuclease-free water (Thermo Fisher) up to 23 µL total volume, and 1 µL of DNA. All samples were run in triplicate in 96-well qPCR plates (Eurogentecs, Seriang, Belgium) using the QuantStudio qPCR system (Thermo Fisher Scientific, MA, USA). The steps of the qPCR protocol were one cycle of initial denaturation at 95°C for 30 s, 40 cycles of denaturation at 95°C for 5 s, and annealing at 60°C for 30 s. The results were analyzed with the equipment software (Connect, Thermo Fisher Scientific).

**TABLE 3:** qPCR primers and probes

Bacterial strain	Forward primer (5′-3′)	Reverse primer (5′-3′)	Probe (5′-3′)	Fluorophore/ quencher	Reference
*S. gordonii* DL1	CTG ATG TCA ACC TGA TTA ACG GCA	GCT TGG TCA GAC CCT GAA AAA TCA	CTT TGA GGG AGA TGC TGT CTA CTC CAT GTA	ABY/QSY	(56)
*S. mutans* UA159	GCC TAC AGC TCA GAG ATG CTA TTC T	GCC ATA CAC CAC TCA TGA ATT GA	TGG AAA TGA CGG TCG CCG TTA TGA A	6FAM/TAMRA	(56)
*A. oris* MG1	GGT GGT CTC CAG CAC TGG G	ATC CTG TGC GGA CGT AAC GC	GGG TGA TGG GCA CCG AGG CGT A	6FAM/QSY	(56)
*N. subflava* DSM17610	AAC GTA TTC ACC GCA GTA TG	TGG AGC CAA TCT CAC AAA AC	AGT CCG GAT TGC ACT CTG CAA CTC G	VIC/QSY	(57)
*V. parvula* DSM2008	CGT TTA GGA ATG AGT ACA GCC GTA	CGG ATG GTG TTG AAG ACC CA	ATT CGT ACT GCT GAA TGT GCG GGA G	VIC/QSY	(56)

### Fluorescence *in situ* hybridization and CLSM

Coupon biofilms samples were washed with PBS (Thermo Fisher Scientific, MA, USA), fixed in 4% paraformaldehyde (Sigma Aldrich, UK) for 2 h at 4°C, washed again with PBS and 1 mL of dehydration buffer (50% ethanol in PBS), and placed at −20°C for 2 h. Subsequently, the samples were washed with PBS and incubated in 1 mL lysozyme solution (1 mg mL^−1^) (Thermo Fisher, USA) for 15 min at 37°C. After another wash with PBS, hybridization buffer (0.9 M NaCl, 20 mM Tris-HCl pH 7.2, 0.01% vol/vol SDS, and 25% vol/vol formamide) (Sigma Aldrich, UK) and 250 ng of the appropriate DNA probe (see Table 4) were added. The biofilms were incubated in the dark covered in aluminum foil for 3 h at 46°C. After incubation, the biofilms were washed with a wash buffer (10 mM Tris-HCl pH 9.0 and 1 mM EDTA) (Sigma Aldrich, UK) and incubated in this buffer at 55°C for 10 min. This step was repeated three times. All biofilms were kept in the dark until imaged with the Zeiss LSM880 confocal laser scanning microscope (Zeiss, Germany). Excitation intensity and any post-processing using the Zen Black software (Zeiss, Germany) were recorded within the software for accurate depiction of signal received.

**TABLE 4:** FISH probes used in this research project^*a*^

Bacterial strain	FISH probe	Fluorophore	Reference
*S. gordonii* DL1	CAC CCG TTC TTC TCT TAC A	Alexa 594	(58)
*S. mutans* UA159	ACT CCA GAC TTT CCT GAC	Alexa 488	(58)
*A. oris* MG1	CGG TTA TCC AGA AGA AGG G	Alexa 555	(59)
*N. subflava* DSM17610	AGT CCG GAT TGC ACT CTG CAA CTC G	Alexa 405	Designed with SnapGene
*V. parvula* DSM2008	CTA ACT GTT CGC AAG AAG GC	Alexa 647	(60)
All	GCT GCC TCC CGT AGG AGT	Alexa 405	(61)

^*a*^ Fluorophores were selected to minimize excitation overlap.

### Live/dead analysis

Biofilms were scraped off the hydroxyapatite coupons and suspended in filter-sterilized PBS (Thermo Fisher Scientific, MA, USA). Cells were centrifuged at 12,000 × *g* at 4°C and resuspended in 1 mL of PBS. The live/dead cell numbers were determined using the LIVE/DEAD BacLight Bacterial Viability Kit (Thermo Fisher Scientific), following the manufacturer’s instructions. Following the staining with SYTO 9 and propidium iodide and incubation in the dark for 15 min, the samples were analyzed on the Attune Nxt Acoustic Focusing Cytometer (Life Technologies, Carlsbad, CA, USA), equipped with a 488-nm laser.

## Data Availability

Data created during this research are available at Newcastle University Research Data Archive at https://doi.org/10.25405/data.ncl.c.6882805

## SUPPLEMENTAL MATERIAL

Appendix spectrum.03713-23-s0001.docx Supplementary data referred to in the main text.

ASM does not own the copyrights to Supplemental Material that may be linked to, or accessed through, an article. The authors have granted ASM a non-exclusive, world-wide license to publish the Supplemental Material files. Please contact the corresponding author directly for reuse.
