# Supplementary material for: Effects of glucose and lactate on Streptococcus mutans abundance in a novel multispecies oral biofilm model
Source: Microbiol Spectr. 2024 Feb 20;12(4):e03713-23. doi: 10.1128/spectrum.03713-23 (PMC10986578; doi:10.1128/spectrum.03713-23)
Supplement: Appendix — Supplementary data referred to in the main text. [file spectrum.03713-23-s0001.docx]

Appendix

Table A1. The composition of AFMC medium, including D-glucose and lactic acid concentrations used in the reactor experiment 1 (high glucose, high lactic acid)

| **No** | **Component** | **Concentration (g L^-1^)** | **No** | **Component** | **Concentration (g L^-1^)** |
| --- | --- | --- | --- | --- | --- |
| 1 | Monopotassium dihydrogen phosphate | 0.44 | 26 | L-lysine.HCl | 0.1376 |
| 2 | Dipotassium hydrogen phosphate | 0.3 | 27 | L-glutamine | 0.005 |
| 3 | Ammonium sulfate | 0.6 | 28 | Nicotinamide | 0.0009 |
| 4 | Disodium hydrogen phosphate | 3.15 | 29 | D-pantothenate | 0.00072 |
| 5 | Monosodium dihydrogen phosphate monohydrate | 2.665 | 30 | Thiamine.HCl | 0.000336 |
| 6 | Sodium citrate | 0.25625 | 31 | p-aminobenzoic acid | 0.00009 |
| 7 | Magnesium sulfate heptahydrate | 0.39 | 32 | Pyridoxamine.2HCl | 0.0010368 |
| 8 | Sodium chloride | 0.01 | 33 | L-glutamic acid | 0.27027027 |
| 9 | Iron (II) sulfate heptahydrate | 0.022 | 34 | L-tyrosine | 0.18018018 |
| 10 | Manganese sulfate heptahydrate | 0.0145 | 35 | L-tryptophan | 0.2 |
| 11 | Sodium acetate | 6.12 | 36 | L-cystine | 0.2 |
| 12 | L-aspartic acid | 0.1 | 37 | Riboflavin | 0.0004 |
| 13 | L-phenylalanine | 0.1 | 38 | Biotin | 0.0001 |
| 14 | L-serine | 0.1 | 39 | Folic acid | 0.0001 |
| 15 | L-proline | 0.2 | 40 | Adenine sulphate | 0.023179937 |
| 16 | L-hydroxyproline | 0.2 | 41 | Guanine | 0.013148283 |
| 17 | Glycine | 0.2 | 42 | Uracil | 0.013148283 |
| 18 | L-methionine | 0.1 | 43 | Calcium chloride | 0.0147 |
| 19 | L-leucine | 0.1 | 44 | Pimelic acid | 0.001 |
| 20 | DL-alanine | 0.1 | 45 | Putrescine (1-4 Diaminobutane) | 0.01 |
| 21 | L-isoleucine | 0.1 | 46 | Thioctic acid | 0.001 |
| 22 | L-threonine | 0.1 | 47 | Cysteine | 1 |
| 23 | L-arginine.HCl | 0.24 | 48 | Inositol | 0.002 |
| 24 | L-histidine.HCl.H_2_O | 0.272 | 49 | D-glucose | 20 |
| 25 | L-valine | 0.1 | 50 | Lactic acid | 12.1 |

**
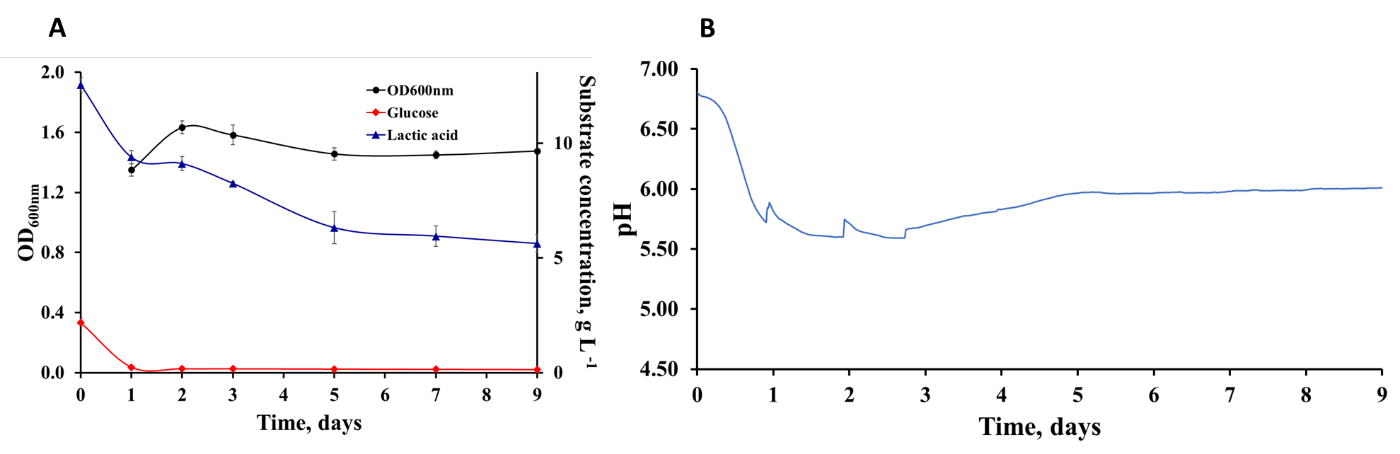
**

**Figure A1***.* Bulk measurements in the CDC coupon bioreactor during the RE 2 (low glucose and high lactic acid, average over 3 replicates). (A) OD_600nm_, concentration of glucose and lactic acid (A); (B) pH.


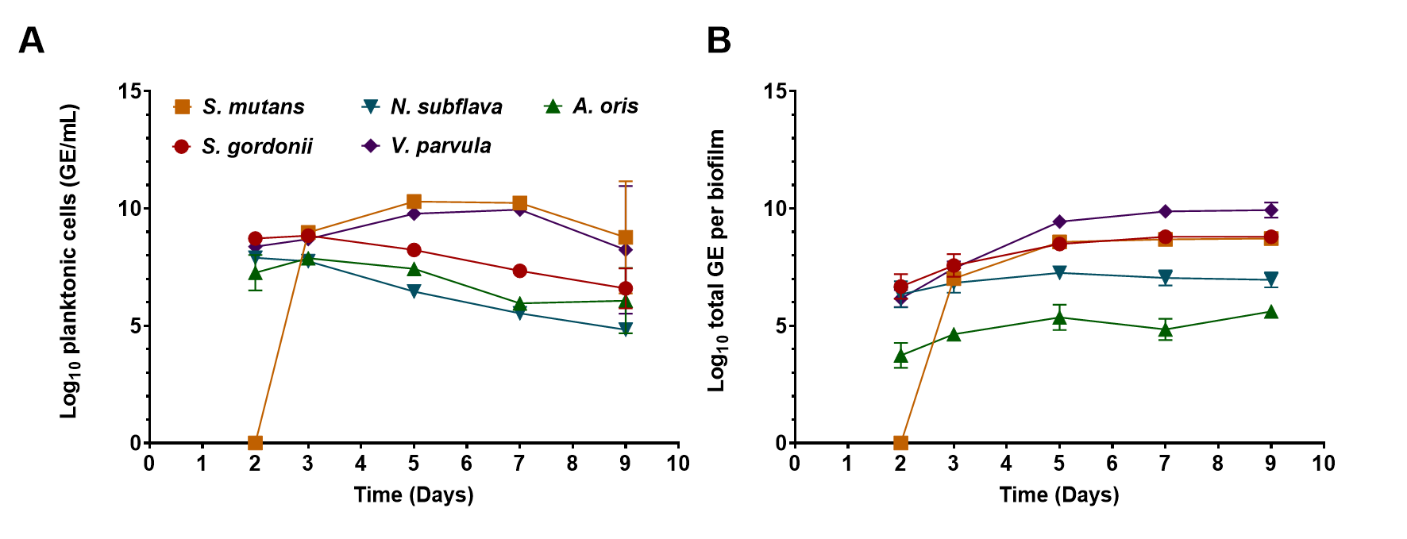


**Figure A2.** Species absolute numbers in the CDC reactor during the RE 2, measured by qPCR. (A) cells in bulk samples; (B) cells in biofilms on hydroxyapatite coupons. Data are expressed as genome equivalents (GE). Data points represent mean values (n = 3) and SD is shown. *S. gordonii*, *A. oris* and *N. subflava* decreased in the bulk fluid between day 3 and day 9, whereas all species were retained in the biofilm.

**
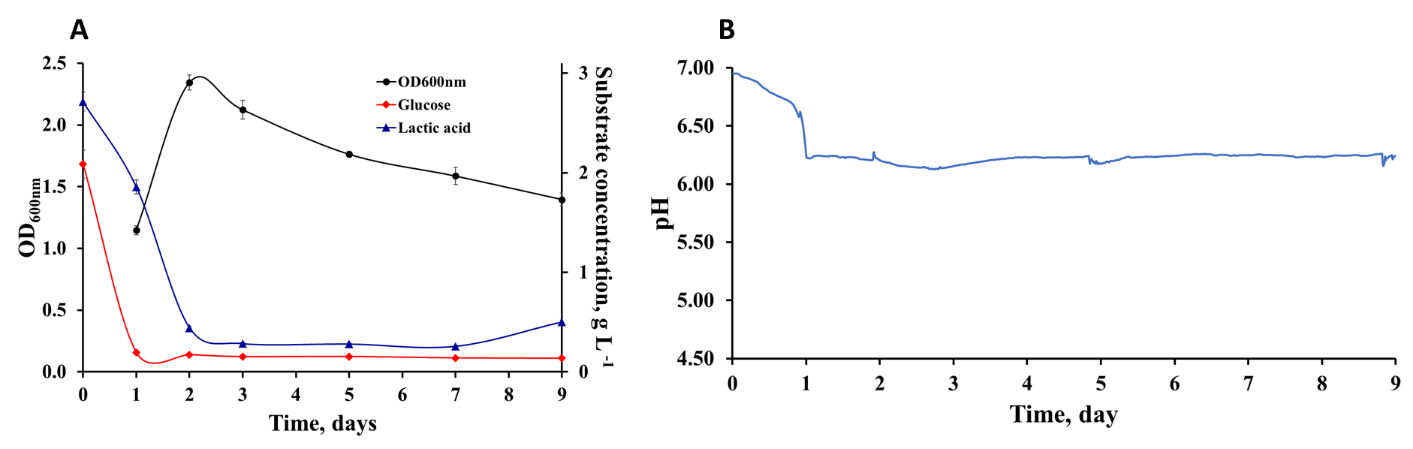
**

**Figure A3***.* Bulk measurements in the CDC coupon bioreactor during the RE 3 (average over 3 replicates). (A) OD_600_nm, concentration of glucose and lactic acid (A); (B) pH


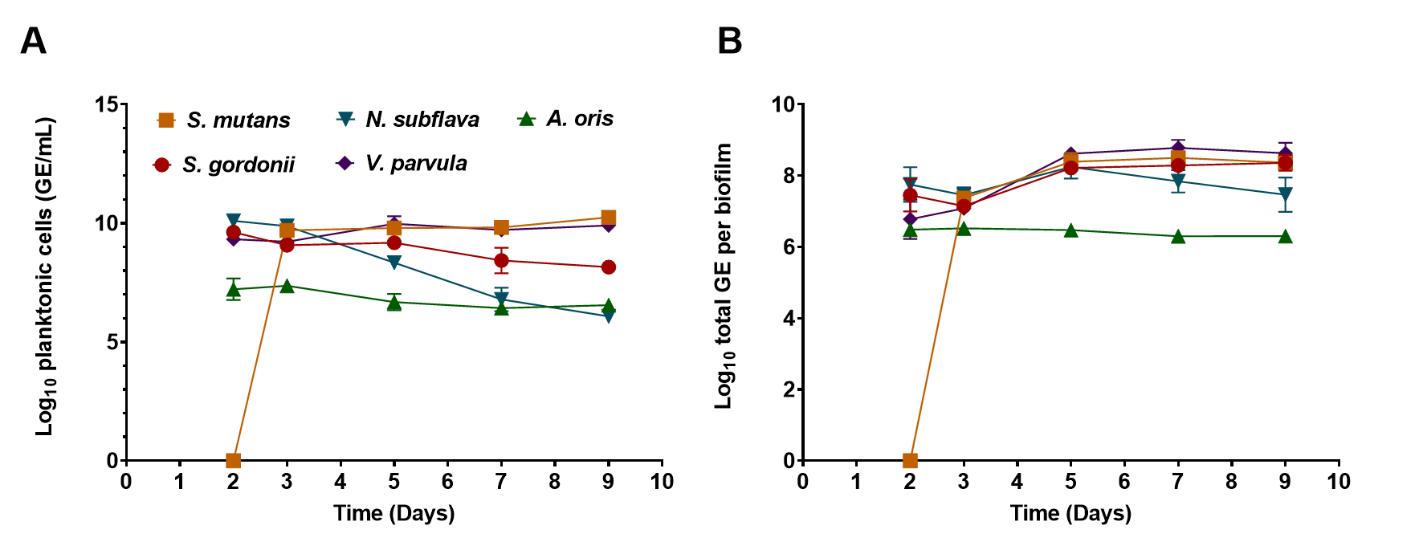


**Figure A4.** Species absolute numbers in the CDC reactor during the RE 3, measured by qPCR. (A) cells in bulk samples; (B) cells in biofilms on hydroxyapatite coupons. Data are expressed as genome equivalents (GE). Data points represent mean values (n = 3) and SD is shown. All species except *A. oris* increased in the biofilm between day 3 and day 5, reaching a relatively balanced biofilm community that was sustained up to day 9.

**
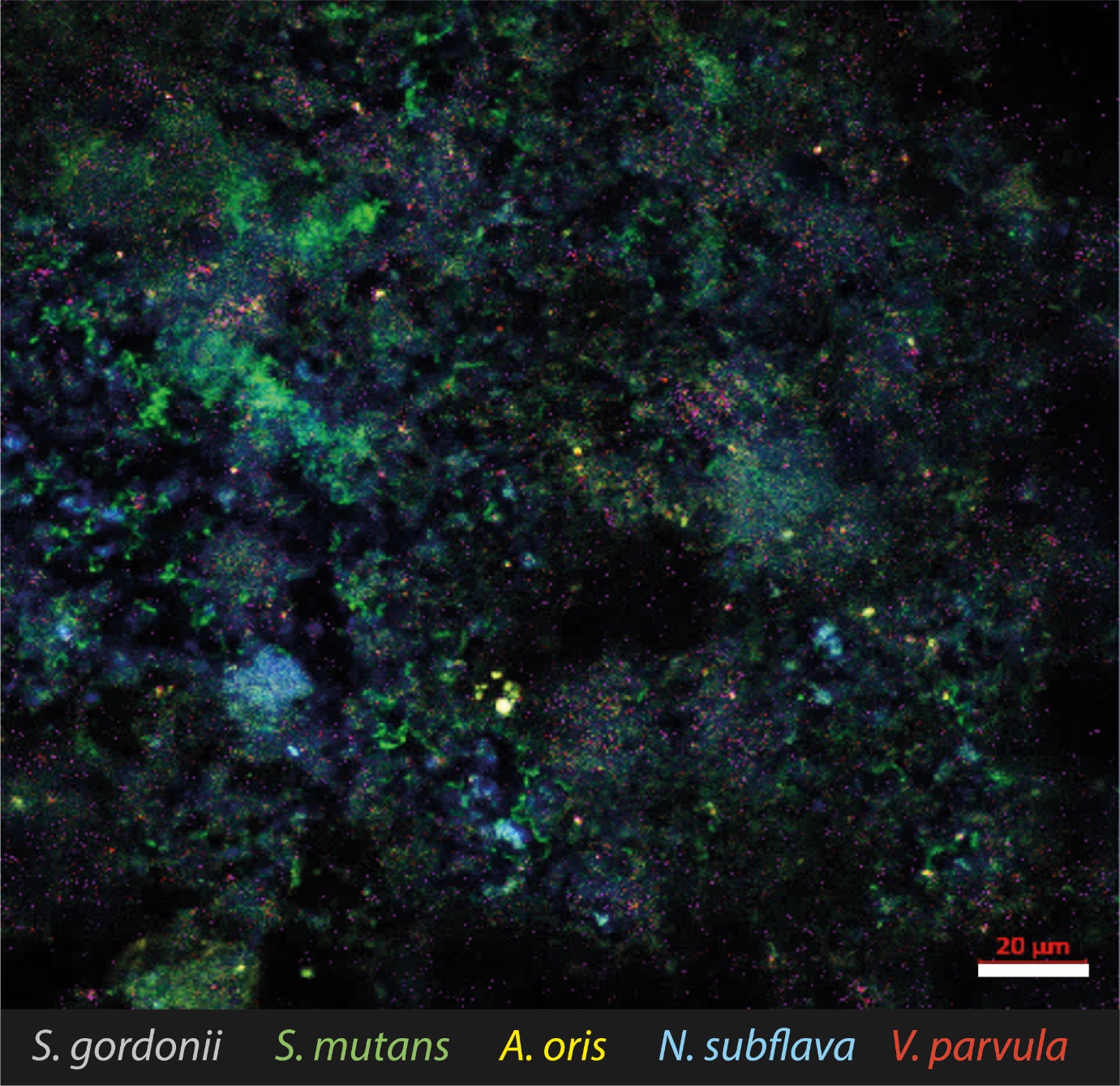
**

**Figure A5** Selected Day 9 FISH-CLSM maximum projection image for RE 3 (low glucose, low lactic acid) showing a relatively balanced composition in the biofilms. Species were labelled with unique FISH probes (described in Table 4) and signals were separated by spectral imaging. Bar = 20 $\mu$m


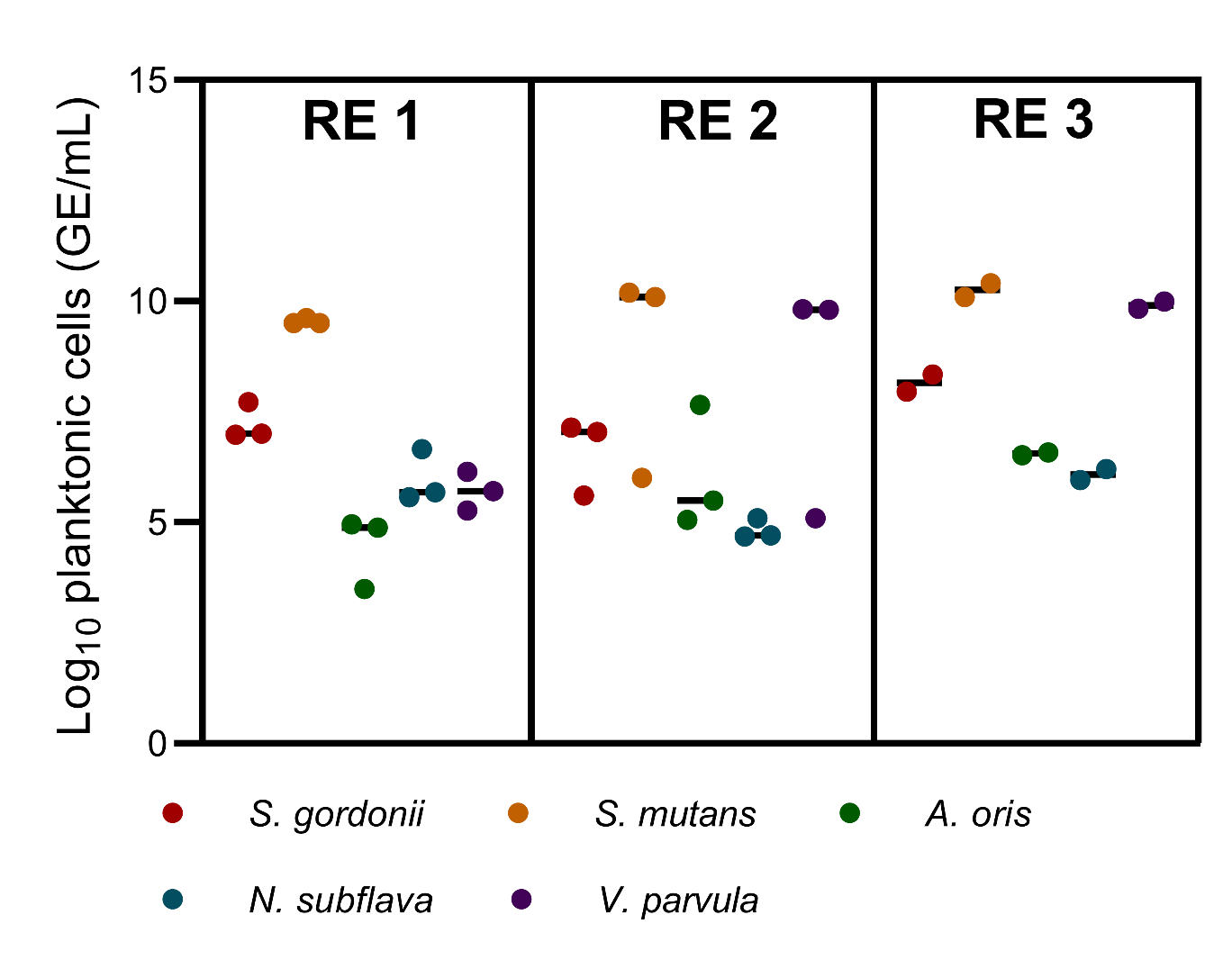


**Figure A6***.* Log_10_ cell numbers in the bulk (qPCR) at Day 9, for all three experimental conditions. *S. mutans* clearly dominated at high glucose concentration (RE 1). At low glucose concentration (RE 2 and RE 3), *S. mutans* and *V. parvula* were the dominant species. Bars show mean values (n = 2-3). GE, genome equivalents.


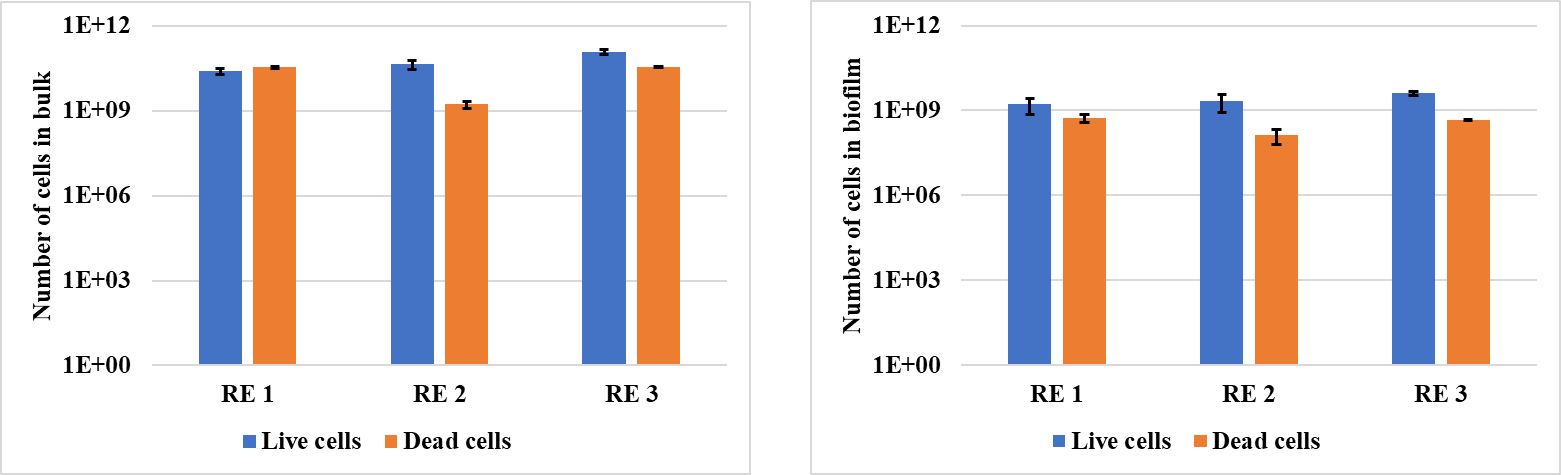


**Figure A7***.* Live/Dead cell numbers in the bulk and biofilm at Day 9, for all three experimental conditions. Viability was measured by Live/Dead staining and flow cytometry. Bars show mean values from 3 independent reactor runs and SD is indicated.
